# A behind‐the‐scenes tour of the IEDB curation process: an optimized process empirically integrating automation and human curation efforts

**DOI:** 10.1111/imm.13234

**Published:** 2020-07-26

**Authors:** Nima Salimi, Lindy Edwards, Gabriele Foos, Jason A. Greenbaum, Sheridan Martini, Brian Reardon, Deborah Shackelford, Randi Vita, Leora Zalman, Bjoern Peters, Alessandro Sette

**Affiliations:** ^1^ Division of Vaccine Discovery La Jolla Institute for Immunology La Jolla CA USA; ^2^ Department of Medicine University of California, San Diego San Diego CA USA

**Keywords:** B cell, curation, database, epitope, T cell

## Abstract

The Immune Epitope Database and Analysis Resource (IEDB) provides the scientific community with open access to epitope data, as well as epitope prediction and analysis tools. The IEDB houses the most extensive collection of experimentally validated B‐cell and T‐cell epitope data, sourced primarily from published literature by expert curation. The data procurement requires systematic identification, categorization, curation and quality‐checking processes. Here, we provide insights into these processes, with particular focus on the dividends they have paid in terms of attaining project milestones, as well as how objective analyses of our processes have identified opportunities for process optimization. These experiences are shared as a case study of the benefits of process implementation and review in biomedical big data, as well as to encourage idea‐sharing among players in this ever‐growing space.

## Graphical abstract

The Immune Epitope Database and Analysis Resource (IEDB) provides the scientific community with open access to epitope data, as well as epitope prediction and analysis tools. Herein, we provide insights into an objective analysis of our data identification, categorization, curation and quality‐checking processes. These experiences are shared as a case study of the benefits of process implementation and review in biomedical big data, as well as to encourage idea‐sharing among players in this ever‐growing space.

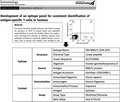

**Abbreviations**
BCR: B‐cell receptor
ChEBI: Chemical Entities of Biological Interest
DST: Data Submission Tool
IEDB: Immune Epitope Database and Analysis Resource
PMIDs: PubMed identifiers
TCR: T‐cell receptor

## Introduction

The Immune Epitope Database (IEDB, iedb.org) is a freely accessible repository of immune epitope data related to epitopes that bind major histocompatibility complex (MHC) or antibodies, are recognized by T cells/T‐cell receptors (TCRs) or are recognized by B cells/B‐cell receptors (BCRs).^1^ The scope encompasses epitopes recognized by humans, other primates, mice and any other species for which data are available. The epitopes can be either linear or discontinuous peptides or non‐peptidic molecules, and their sources range from microbes to allergens, autoantigens and transplantation antigens.

The founding principle that inspired the inception of the IEDB in 2003^2^ was to facilitate immunological research by providing the community with a searchable resource housing not only the sequence and chemical structures of epitopes, but also the actual primary data and metadata associated with them. With regard to breadth and depth of epitope data, no comparable resource existed at the time, and the IEDB remains the most comprehensive epitope data resource today. The information curated in the IEDB is derived from the scientific literature catalogued in PubMed,^3^ and from direct data submissions from users, or more often from various NIH‐sponsored research efforts. Accordingly, for each epitope, it is necessary to extract the scientific details defining the assays in which the epitopes are defined and studied. The same epitope might be studied in multiple publications or submissions, and the same epitope is commonly tested in multiple assays, sometimes with different outcomes (e.g. a virus‐specific antibody might bind in an enzyme‐linked immunosorbent assay format but not neutralize the live virus). Such nuances in the assay parameters accompanying the epitopes are objectively made available to view, based on the end‐user’s defined query.

A highly specialized expert curation process is pivotal to ensuring the consistency of such highly contextual data. This is achieved by Curators who review the data, systematically extracting and depositing the relevant information into a computer‐operable format.^4^ For instance, the relevant information is not confined to a single location of the manuscript. Rather, the structure of the epitope as well as the contextual assay details may be reported in the methods section, while the data and interpretation of the results are often found in figures, table and text. The Curator is therefore challenged to faithfully synthesize these disjointed elements of a publication into a concise format.

Here we present an account of the processes that the IEDB team has developed, periodically reviewed and iteratively improved to meet these challenges and to define a formalized workflow related to curation of this highly contextual immunological scientific literature. The purposes of a formalized workflow are to achieve process documentation, facilitate curation, ensure consistency, and identify bottlenecks and opportunities for further gains in efficiency and accuracy.

**Figure 1:**
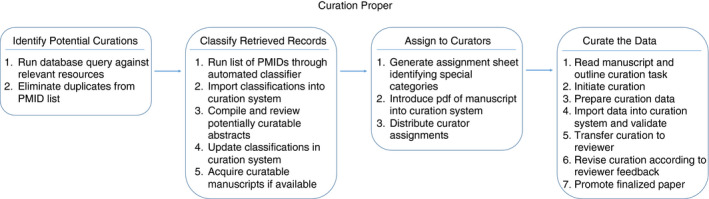
The IEDB curation process consists of four high‐level steps, each with its own specific sub‐steps designed to ensure data quality and consistency.

For the sake of analysis, the curation process is divided into four high‐level steps (Fig. 1), including the identification of the relevant data, the classification of these data and the assignment of the data to Curators, followed by the actual curation of the data by the Curators. Each step is further composed of sub‐steps or sub‐processes, and clearly identifies an input, an output (typically representing the input of the following step), and an ‘actor’ who performs the activities detailed in each step. In addition, we record the frequency by which each step occurs, and any potential improvements.

## Curation proper

### Query relevant resources for potentially curatable data

The first step in the curation process involves running a query on the PubMed database to generate a list of retrieved records. The query script is formulated to be purposely broad in order to capture as many potentially relevant papers as possible. This query is run on a biweekly basis by a Document Specialist. The output is a list of PubMed identifiers (PMIDs), to be evaluated in further steps. The query script itself is updated as the need arises, to include new terms or revise its content, as the IEDB scope and the nature of the scientific literature evolves. This query channel retrieves the vast majority of the data that are ultimately incorporated into the IEDB.

In parallel, also on a biweekly basis, the Document Specialist queries the Protein Database (PDB; rcsb.org)^5^ to generate a list of published literature reporting three‐dimensional (3D) structures of immunological interest. This query generates a list of PMIDs and their corresponding PDB IDs and relevant chains. The PMIDs from this list are then compared with the records stored in a centralized curation tracking system to identify those manuscripts that have yet to be evaluated; the unevaluated references are then sent to a Curator to be examined for structures of immunological interest. The PDB classifier runs regularly, extracts the latest PDB IDs, classifies them as an immune receptor (and type) or not, then outputs a file that includes the PMID linked to the PDB. This list of PMIDs is then merged with the list described above, and subsequently moves through the pipeline to the classification step described in the following section. Finally, the IEDB personnel also run specialized queries to identify any paper containing tetrameric staining reagents that might have been missed by the PubMed and PDB queries. The use of tetramer reagents to identify, isolate and characterize epitope‐specific T cells has seen tremendous growth in the past decade, and it is therefore important for the immunological community to have a catalogue of these reagents to identify those that may be applicable for their own research needs. Here as well, the results of the tetramer query are compared with the records already stored in the curation tracking system to eliminate redundancies.

### Classify retrieved records

The outcome of the queries above is a combined list of potentially relevant PMIDs. An automated document classifier^6^ next processes the list of retrieved records to generate a binary curatability assessment, referred to as ‘curatable’ and ‘uncuratable’. Furthermore, the classifier assigns the potentially curatable PMIDs to broad subject matter categories and subcategories (i.e. Allergy; pollen or Infectious Disease; poxviruses). These determinations are used to prioritize and track curation activities. At this stage, the Document Specialist updates the curation systems to reflect curatability and category assignments. Specifically, the Document Specialist extracts from the curation system a list of potentially curatable PMIDs. This list is then used to generate an ‘abstract book,’ in either a paper or electronic format, which contains the authors, title, reference information and abstract for each potentially curatable PMID.

The Document Specialist and a Senior Immunologist next jointly review the abstract book for accuracy of the classification and for the likelihood of curatability. The result of this activity is an annotated abstract book. The Document Specialist at this point checks availability of potentially curatable PMIDs and acquires available manuscripts via PubMed. If necessary, authors are contacted for their manuscripts if the reference is not freely available online. Author contact becomes necessary for approximately 20% of the manuscripts. We have, therefore, automated the extraction of the corresponding author’s email address, which is then used to draft a personalized request email to the author. Full PDF files of available manuscripts are obtained and the curation system is updated to reflect availability. On a biweekly basis, the Document Specialist checks whether previously unavailable manuscripts have become available, first by checking PubMed and then by checking the University of California, San Diego library.

Once the manuscripts have been checked for availability and acquired, the Document Specialist adds the output of the queries to the curation tracking system. The output includes all manuscripts that were not previously in the curation system that have been retrieved by the current query, including those that the automated document classifier or the Senior Immunologist determined to be uncuratable. The tracking system is also updated to reflect any previously unavailable manuscripts that have become newly available, if applicable.

On an annual basis, the automated classifier is re‐trained, taking advantage of the updated curatability and category assignments generated to date.

### Assign papers to curators

Using the full PDF files of available manuscripts, the Document Specialist next generates an assignment sheet and identifies papers requiring a differential curation process, namely those that appear to contain non‐peptidic epitopes, or structural and/or immune receptor (BCR or TCR) sequence information. The curation systems are then accordingly updated to reflect curatability and category assignment revisions, if applicable. The full PDF files of available manuscripts are then introduced into the internal curation system, a web application, where Curators access manuscripts and manually input the data contained within each. The Document Specialist uses the assignment sheet to distribute curation assignments to individual Curators, by means of emailing the specific Curators with individual assignments.

### Curation of journal articles

**Figure 2:**
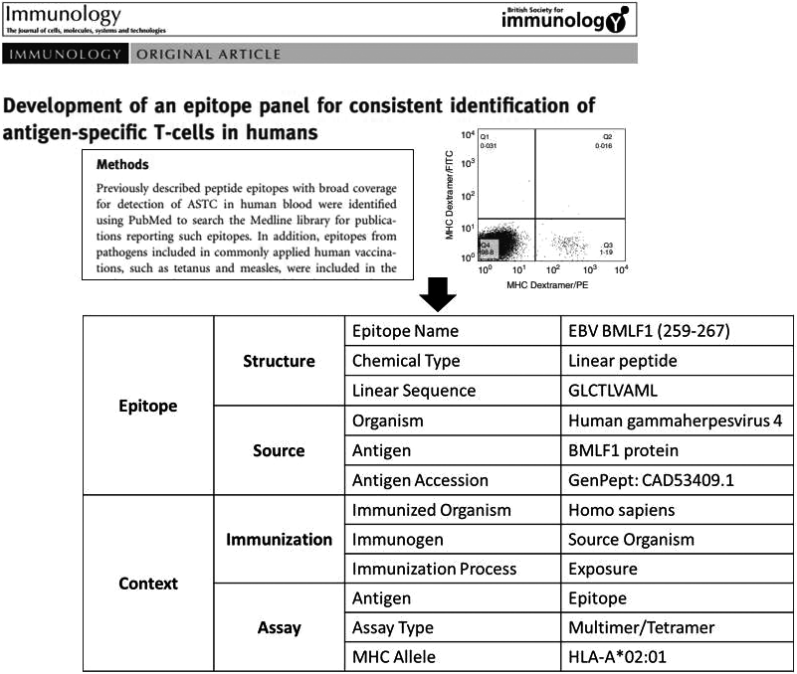
The assay‐centric, contextual representation of epitope data is rooted in an ontologically based curation system enabling the Curator to extract and input data from text and figures into defined data fields.

The expert curation process is primarily completed using the web‐based curation system.^7^ Within the curation system, each Curator has an assigned account that enables the Curator to manage a personal queue of manuscripts with statuses ranging from pending curation to curation completed. In this way, the status of each curation can be tracked in real time. Next, the Curator reads the paper and scopes the curation task, generating a plan to curate the data in the paper. This often involves review of cited references, contacting authors and other activities that result in identifying curatable data. As a result, curation data are entered at the data field level into a curation system with an ontological architecture (Fig. 2), and the Curator marks the paper as ‘initial curation done.’ The papers containing BCR/TCR sequence information or structural data are flagged as applicable. An annotated hard copy of the manuscript and a report of the curated data are both printed, as well as a cover sheet that is filled in to track the review progress. This process is described in a later section.

### Curation of Data Submission Tool records

In the next few sections, we describe special processes that are put in place to curate the papers that are flagged because they require the use of the Data Submission Tool (DST), contain BCR/TCR sequence information, contain structural data, or are published papers with related data that are directly submitted to the IEDB.

The DST is a web‐based portal (http://dst.liai.org) designed to facilitate the upload of large‐scale epitope data directly into the IEDB curation interface. From there, the data can be further edited, expanded and ultimately reviewed before release to the IEDB website for public consumption. The DST was specifically designed to assist in the curation of records that contain numerous repetitive data (like the isolation of thousands of different peptides eluted from the same MHC, using the same technique), and uses a spreadsheet format. The DST is also used to process direct submissions to the IEDB database from the scientific community. The initial stages are the same as described above, but in addition the Curator obtains the epitope sources using a web‐based Mass BLAST tool developed specifically by the IEDB. This tool enables the Curator to efficiently obtain the GenBank sources in which large batches of peptides are completely conserved (up to 10 000 peptides per run) by providing the peptide sequences and source organisms as inputs. This tool eliminates the need to tediously BLAST individual peptides, or even small batches of peptides, in order to obtain these GenBank identifiers, which are a required parameter for the majority of the epitopes catalogued in the IEDB. The Mass BLAST tool output can also be easily integrated into the DST template spreadsheet, which is subsequently uploaded and validated using the automated validation rules that are integrated into the DST. The rest of the curation then proceeds as described in the preceding section.

The DST spreadsheets are updated and optimized on an ongoing basis by IEDB staff. Field‐level values that are chosen by the Curator (e.g. cell types) are refreshed on a weekly basis to maintain synchronicity with the main IEDB curation website. Updates to the breadth of the fields themselves are also performed periodically to accurately represent the published data. All updates are accomplished by a combination of manual requests for expanded data fields/values and automated processes that iteratively refresh the templates.

### Curation of non‐peptidic, 3D‐structure and TCR/BCR papers

The curation of papers containing non‐peptidic epitopes also requires some specialized steps.^8^ In these cases, the Curator first identifies if any non‐peptidic molecules that are not already present in the IEDB will be needed for curation. To this end, the Curator submits the non‐peptidic structures to a new term request template, used for all ontology term requests, including organisms and proteins. An IEDB staff member assigned to this task then determines if the structure already exists within the IEDB data tables. If it does, the appropriate Chemical Entities of Biological Interest (ChEBI)^9^ identifier is entered into the template. If the structure is not already available, ChEBI is searched for a corresponding structure. If one can be identified on the ChEBI website, it is introduced into the IEDB data table and made available for the Curator to use. If no appropriate ChEBI structure can be identified, internal IEDB identifiers are used instead. When this is done, a structure name is entered for the internal identifier and each structure is assigned an immediate parent within the ChEBI tree, specifically adapted for IEDB use. The rest of the curation then proceeds as described in the preceding section. The IEDB‐adapted ChEBI tree was created by removing all nodes from the entirety of ChEBI that have no data‐points within the IEDB. With each weekly build of the IEDB, this process of pruning is performed again, to account for any newly utilized terms.

In terms of curation of 3D structural papers, the process starts with the list of PDB records identified as described above. This list is inspected to generate Chain IDs for ligands, receptors and MHC molecules. An IEDB Curator will typically access associated PDB and NCBI structure files to obtain Chain IDs and any possible PDB identifiers for non‐peptidic ligands, modified residues and glycan components. To address different types of structural records, the Curator determines which chains are part of the biological complex and adds the relevant BCR/TCR chain IDs to the molecule finder, enabling them to be used in curated fields of the database. Next, the Curator with the aid of a 3D Contacts Tool embedded into the curation interface, will enter PDB and chain IDs and peptide/ligand sequences into the 3D complex fields to calculate the contacts between each chain. The calculated epitope residues or the specified ChEBI structure defined in this process are then entered into the epitope fields. If the ChEBI structure is not already present within the IEDB, the steps described above for non‐peptidic structures are followed. From this point on, the standard process for the entering of curated data in the system is followed.

### Curation of dual submissions

In certain cases, the same primary data are contained in papers published in the scientific literature and also in direct submission made to the IEDB, typically but not exclusively as related to the work of large‐scale epitope identification contracts. To date, the IEDB has received 32 of these data submissions. The data curated in the direct submissions is largely overlapping with the published data, but typically the direct submissions are more comprehensive and may, for example, also include negative data, which are less commonly included in published reports.

In these cases, if the manuscript is published first, a Curator contacts the author to request raw data and/or submission files. These files are then transferred into the DST spreadsheet or the author submitted data are edited appropriately. The rest of the curation process proceeds as described for the ‘regular’ papers.

### Review and approval of curated papers and submissions

The IEDB curation process incorporates a peer‐review of the curation performed by its staff, to ensure accuracy and facilitate consistency across different curations. As a first step in this process, the Document Specialist assigns a reviewer and delivers to the reviewer a printed hard copy and the previously completed cover sheet. The reviewer then reviews the curation record and returns feedback on the cover sheet to the Curator. This process can be iterative, much like review of a paper submitted for publication, until a consensus is reached. The revised curation is then approved and marked as ‘final’ in the curation system.

In the case of papers containing TCR/BCR sequences, additional review and data‐entering steps are required.^10^ After the curated data have been peer‐reviewed and finalized, all assays that tested a specific antibody or TCR, for which any sequence information is known, will be joined with additional receptor sequence information. Curators fill out an additional receptor template with all available receptor information, including full‐length nucleotide or protein sequence, curated complementarity‐determining regions 1, 2 or 3 sequences, and gene usage, depending on what is available in each manuscript. The receptor sequence template is then manually reviewed for errors and, after peer‐review, undergoes automated validation and calculations. Validation is performed by a Bioinformatics Specialist using Python scripts to check for non‐amino acid residues in sequence fields, as well as conforming gene name formatting. When errors are identified, the Curator refers back to the manuscript to make corrections. Concurrent with the validation process, additional data fields are calculated to include complementarity‐determining regions 1, 2 or 3 and V domain. These calculations are only performed when a full‐length nucleotide or protein sequence is available and uses ANARCI software, as previously described.^10^ Once all errors are corrected and all calculations are completed, the template is loaded directly into the receptor data tables. The data are then joined with the curated assays as part of the following weekly build, and becomes publicly visible.

## Analysis of curation processes

### Quantifying the effort required for each step, to identify bottlenecks and optimize curation effectiveness

As mentioned above, we continually seek to appraise the efficacy of the processes described herein. Therefore, we quantified the effort required to perform the various tasks described above in order to identify which activities might be most onerous and thereby could yield most gains in effectiveness if restructured, automated or otherwise streamlined. To this end, we interviewed and compiled input from all IEDB staff involved in the curation processes described above. Currently, the IEDB curation staff is comprised of nine Curators, and a single Document Specialist. Each staff member was asked to quantify the percentage of their time devoted to each of the tasks. It should be noted that several tasks are shared by multiple IEDB staff and each IEDB staff member participates in multiple tasks, and also works on non‐curation tasks.

**Figure 3:**
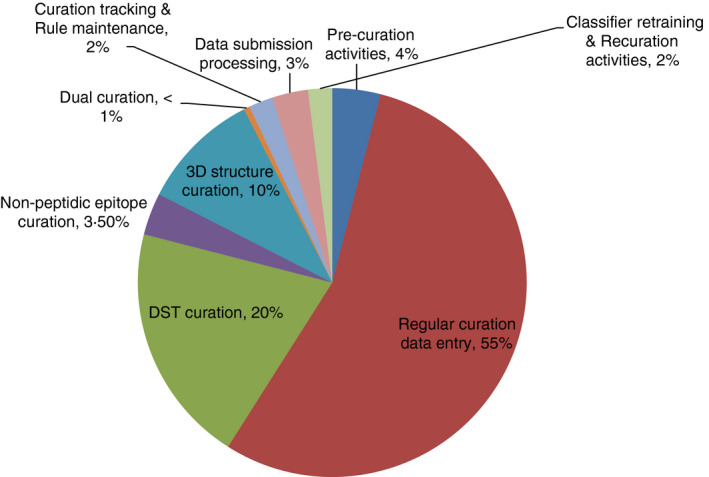
The distribution of effort required for the major steps in the curation workflow, based on compiled input from IEDB staff. This analysis was used to target steps for process optimization.

This analysis indicated that querying and classifying potentially curatable records, scanning for curatability and scope, and assigning papers to Curators accounted for about 4% of the total curation effort. Regular curation accounted for about 55% of the effort in total, with approximately half (27%) of that effort devoted to actual data entering, and 16% of the effort dedicated to peer‐review and curation record revisions. It is important to note that approximately 70% of the manuscripts retrieved from our queries ultimately remain curatable after the various screening steps, with the remaining 30% deemed not to contain data, or to contain data irrelevant to the IEDB scope. Curating DST papers accounted for about 20% of the total curation effort, with again data entering and peer‐review being major activities. Activities specific to curation of non‐peptidic papers accounted for approximately 3·5% of the total curation, with identification of the molecular structures and representing them through identifiers in ChEBI^8^ being a major task. The specific aspects of curation of 3D and receptor papers accounted for about 10% of the effort. Specific attention required for dual (paper and direct submissions) curations accounted for <1% of the total, and about 2·0% was required to track progress and update curation rules. About 3% of the total effort was required for activities related specifically to direct submissions. Finally, about 2·0% was required for retraining the classifier and recuration activities (Fig. 3).

### An analysis of the peer‐review process and outcomes

The data presented above indicate that a significant fraction of the total curation effort is absorbed by the peer‐review process of curated records. Here we undertook an analysis of the process to examine whether it could be streamlined or optimized, so realizing significant gains in overall efficiency. A total of 97 previously curated and reviewed papers was included in the analysis; of those, 72 (74%) had issues identified in the review process, while 25 (26%) had no or very minor issues. A total of 192 issues were identified; of which 10 (5%) were typos (misspellings, punctuation, copy/paste errors) and 6 (3%) were duplications (redundant epitopes or assays). These problems could be corrected by automated ‘prevention’ strategies such as spell check and automated validation,^11^ and implementation of these strategies was initiated as a result of the present analysis.

Of the remaining issues, 102 (53%) were curation errors, identified during peer‐review, and 74 (39%) involved missed data (data that were present in the manuscript but not curated). Approximately half of these were characterized as ‘major’ errors involving missing assays and epitopes, different outcomes and different epitope specificity. The remaining half comprised errors where a curation rule was violated.^12^ We also considered how prevention of these errors could be prioritized and errors could be avoided. Major errors are important and should be avoided by additional caution being exercised. In the case of rule errors, we noted that these errors might reflect problems with rules that could be better clarified, or if deemed unnecessary, retired altogether.

The assessment of missed data evaluated what specific type of data is being overlooked. We found that age, gender, geolocation, MHC restriction, MHC types present, number of subjects tested/responded and required assay comments were typical examples of missing data. Although additional care during primary curation would help to prevent these issues, it was also noted that these records were not *per se* wrong, but simply incomplete and therefore a less serious problem than outright errors, in which incorrect data were entered.

We also noted that the number of near‐perfect papers depends both on the type of paper (complexity) and sometimes on the Curator. Easier/simpler papers (fewer epitopes/assays, all same subjects/assays, 3D structure papers) are more often perfect compared with more difficult ones. Additionally, each Curator has a ‘batting average.’ We reasoned that peer‐review of some papers could be replaced by a ‘spot check system,’ where the goal would not be to ensure quality of all papers *per se*, but rather to monitor issues and correct them. Upon further discussion, it was generally agreed that adding this layer of ‘review of the review’ would be cumbersome and would not result in overall gains in efficiency, while decreasing the overall data quality. Alternatively, we envisioned that it would be desirable to develop an automated system where the type of errors would be tallied and compiled over time (based on monitoring which fields are modified upon review), to provide an additional tool to manage the overall curation process and its quality. All Curators undergo an extensive 8‐week training program, but we hold ongoing team training sessions to review our guidelines, and to introduce new guidelines. The results of these automated quality controls could be integrated into such sessions as a further means of training. In terms of a more practical area to improve the peer‐review efficiency, we are also planning to implement a collaborative, electronically based review platform in order to remove the need to print and physically exchange documents.

## Discussion

The establishment of the IEDB represented an unprecedented expert curation undertaking, aimed at creating a repository of published and unpublished epitope data of immense scale and previously unavailable depth. To date, the resource has grown to house in excess of 650 000 epitopes and 2 000 000 assays. At the core of the project’s success are well‐defined workflow processes established during the IEDB’s nascent phases. As custodians of these data, we recognized the importance of implementing rigorous processes to ensure that we meet the data quality and quantity expectations of our user base.

The importance of having such processes in place, especially in the rapidly evolving immunology field, is undeniable. As discussed above, we continue to succeed in precisely extracting relevant publications from PubMed’s more than 30 million indexed publications, categorizing and prioritizing each, systematically curating and reviewing the data within them, and making the data publicly available, all within a 6‐ to 8‐week cycle. This serves the scientific community with easy access to current data to help drive immunological studies and progress.

In addition, we have demonstrated the real‐time adaptability of such processes. In fact, historical experiences have necessitated ‘stress‐tests’ of our workflows, and the outcomes have substantiated their value. For example, during the 2009 swine‐origin H1N1 influenza virus outbreak, our curation processes proved effective as we were well positioned to pivot and adapt our efforts to rapidly and comprehensively capture all outstanding influenza‐related data in the published literature. In so doing, we quickly compiled the largest global repository of Influenza epitope data, which we subsequently analyzed to demonstrate that pre‐existing immunity to swine‐origin H1N1 influenza virus could exist in the human population.^13^ This rapid dissemination of information is a powerful example of the utility of the IEDB, and is directly attributable to the flexibility of our process infrastructure. We recently repeated a practical application of the IEDB with coronavirus in light of the emerging pandemic,^14^ and anticipate the opportunity to again adapt our processes to focus on the imminent publication of important coronavirus epitope data.

Despite the proven value of the IEDB processes, we recognize the importance of periodically reviewing and fine‐tuning them to optimize returns. Our recent analysis of the IEDB peer‐review process, for example, is a prime case study illustrating that establishing quantifiable workflow processes can be leveraged to stringently evaluate their efficacy, and to empirically highlight areas that can be targeted for optimized efficiency. We have identified bottlenecks in our review process, and expect a rise in throughput as a result of addressing these inefficiencies. We share the experiences gained from the IEDB with the firm belief that these outcomes can be broadly applied to benefit other similar projects, especially those dealing with large‐scale data management for which the existence of effective and efficient processes represents critical conditions of success.

Furthermore, another motivation behind this work is user engagement. As an NIH‐funded public resource, we continually strive to engage our users for their input as a means of optimizing our resource. Our ultimate metrics of success are positive user experiences, and the high usage numbers that result. As such, the evolution of the IEDB has been driven by cycles of soliciting user feedback and iteratively refining the IEDB accordingly. Here, for the first time, we are expanding this effort beyond seeking feedback about the finished product alone, but also about the intricacies of the methods and procedures employed to produce the final product itself.

Our rationale for this is twofold; first, we consider our users stakeholders in the resource and as such we feel they should be afforded full transparency in terms of the *how* as well as the *what*. Perhaps having this insider perspective will further enhance the user’s appreciation for the complete capabilities of the IEDB resource and thereby serve to further enhance the user experience. For example, although originally conceived as a resource primarily focused on the epitope and its contextual assay information, the IEDB has grown in recent years to become a prime repository of corresponding BCR and TCR sequence data. Describing the processes by which we identify and extract BCR and TCR data will also heighten user awareness of its availability in the IEDB and promote the user’s ability to leverage this feature to drive discovery and understanding in this growing field. Second, just as we utilize user feedback as a springboard for improvement of the external face of the IEDB resource, it is our hope that sharing these insights will inspire equally valuable user feedback about our internal processes as well. Therefore, we invite constructive criticism of the processes described herein in order to initiate an important open dialogue from which we can further improve our internal processes, and ultimately deliver an improved resource to our users.

Others in the biocuration domain have published introspective reviews of their expert curation workflows.^15^, ^16^ This, to a major extent, is reflective of the increasing importance of expert curation across biological disciplines, and the need to optimize such workflows to maintain pace with the data growth. Although expert curation is often considered the reference standard for data curation, several recent reports have highlighted the budgetary challenges that inevitably limit such efforts.^17^, ^18^ Compounding these limitations is the exponential growth in published data. Together, these factors have led to increased utilization of text mining and community curation approaches.^19^, ^20^, ^21^, ^22^, ^23^ In addition, the NIH Big Data to Knowledge (BD2K) initiative^24^, ^25^, ^26^ has been established to maximize the collection, organization and dissemination of biomedical big data. Nevertheless, there remain significant gains to be made by promoting a discourse around the best practices of expert curation supplemented with automated processes. Hence, we envision not only engaging our user base, but also our peers in the biological knowledge‐base realm to bring to fruition a mutually beneficial exchange of ideas that will ultimately lead to more effective data sharing and research infrastructure.

## Disclosures

The authors declare no conflicts of interest.
